# Myeloid dendritic cells and periodontal disease association: integrated study of single-cell sequencing and Mendelian randomization analysis

**DOI:** 10.3389/fimmu.2024.1522281

**Published:** 2025-01-03

**Authors:** ChengJi Shi, XinYi Ou, XiaoXu Lei, LiJuan Huang, ShuHao Xu, Wei Li, Xi Zhao

**Affiliations:** ^1^ Department of Stomatology, The People’s Hospital of Deyang City, Deyang, Sichuan, China; ^2^ Nanobiosensing and Microfluidic Point-of-Care Testing, Key Laboratory of Luzhou, Department of Clinical Laboratory, The Affiliated Traditional Chinese Medicine Hospital, Southwest Medical University, Luzhou, Sichuan, China

**Keywords:** eQTL, GWAS, Mendelian randomization, myeloid dendritic cells, periodontal disease, single-cell sequencing

## Abstract

**Background:**

Periodontal disease is a widespread inflammatory condition that compromises the supporting structures of the teeth, potentially resulting in tooth loss if left untreated. Despite advancements in therapeutic interventions and an enhanced understanding of its pathophysiology, emerging techniques such as single-cell RNA sequencing (scRNA-seq) and Mendelian randomization (MR) present new opportunities for precision medicine in the management of periodontal disease.

**Methods:**

Data derived from the GSE152042 dataset underwent rigorous quality control, normalization, and dimensionality reduction using Seurat and the MonacoImmuneData framework. Marker genes were identified to delineate subgroups for subsequent analysis utilizing CellChat and ClusterProfilerR. MR analysis of the expression quantitative trait loci (eQTLs) for these genes was conducted to determine causal relationships with periodontal disease, leveraging data from the IEU Open GWAS project.

**Results:**

Single-cell analysis revealed distinct immune cell subtypes and indicated an increased presence of myeloid dendritic cells (mDCs) in patients with periodontal disease. MR analysis identified twenty-six significant genes, with LIMA1 (LIM domain and actin-binding 1) demonstrating a robust causal association with the progression of periodontal disease. Gene ontology and Kyoto Encyclopedia of Genes and Genomes analyses highlighted crucial pathways involved in periodontal inflammation and tissue destruction. Visualization at the single-cell level elucidated the role of LIMA1 in disease progression, alongside differences in cell communication dynamics between LIMA1-positive and -negative populations.

**Conclusion:**

This study underscores the utility of scRNA-seq and MR in elucidating essential factors in the pathogenesis of periodontal disease, thereby reinforcing the necessity for targeted therapeutic strategies. The identification of LIMA1 as a pivotal gene in periodontal disease progression opens new avenues for precision medicine approaches, potentially enhancing treatment efficacy and patient outcomes in periodontal management.

## 1 Introduction

Periodontitis is a chronic inflammatory disease with multiple contributing factors, linked to the buildup of dental plaque, and is characterized by the gradual destruction of the structures supporting the teeth, including the periodontal ligament and alveolar bone (1, 2). Periodontal disease affects approximately 11% of the global adult population—around 743 million individuals with severe periodontitis—is influenced by various risk factors, including genetic predispositions, environmental exposures, and unhealthy lifestyles (3, 4). While there has been progress in understanding the mechanisms of periodontal disease, its complexity remains incompletely elucidated, and treatment prospects continue to face challenges. With the development of treatment strategies for periodontal disease, researchers have shifted from non-specific antibiotics to more targeted approaches aimed at modulating immune responses and promoting tissue regeneration (5, 6). However, due to the heterogeneity of periodontal disease, variations in age of onset, differences in severity, the transmission of bacteria between individuals, and the involvement of multiple pathogens, it becomes difficult to clarify the role of genetic factors in the pathogenesis of periodontal disease. Furthermore, many patients often show poor responses or experience adverse reactions during treatment, complicates the treatment process (7). Therefore, there is an urgent need to develop precise intervention strategies based on individual patient characteristics to advance the field of precision medicine.

Identifying the molecular features and cellular changes in periodontal tissues has always been challenging, primarily due to the presence of diverse cell populations. Gingival tissue comprises various cell types, including epithelial cells, infiltrating immune cells, fibroblasts, and endothelial cells (8). The interactions among these cells collectively maintain tissue homeostasis and influence the onset and progression of periodontitis. Traditional bulk RNA sequencing and microarray technologies can only provide average expression levels for the samples, and due to the coexistence of multiple cell types in gingival tissues, these methods fail to reveal information specific to particular cell types and cellular heterogeneity. Recently, the introduction of scRNA-seq technology has made it possible to identify gene expression profiles at the single-cell level with unprecedented accuracy. Compared to cell sorting and subsequent transcriptomic analysis, scRNA-seq greatly expands the range of cell characterization and subtyping because it does not rely on pre-defined differentiation cluster (CD) proteins and provides high-resolution cellular gene profiling. This unbiased analysis of cellular changes can deeply unveil the entire periodontal tissue ecosystem, including the mechanisms of cell signaling and intercellular interactions (9).

MR serves as a powerful analytical tool for inferring causal relationships between genetic variants and complex traits or diseases (10). MR utilizes the random assortment of alleles at conception as a natural experiment, providing a robust framework to validate the functional relevance of candidate markers identified through scRNA-seq (11). Additionally, it circumvents confounding factors and reverse causation inherent in observational studies. By integrating genetic instruments associated with potential markers, MR analysis can elucidate the genetic determinants of periodontal disease risk and progression, facilitating the translation of molecular discoveries into clinical applications (12).

By combining cutting-edge scRNA-seq with MR analysis, this integrative approach has the potential to enhance our understanding of periodontal disease. It facilitates the exploration of genetic variation’s impact on the expression of various cell subpopulation markers in periodontal tissue through single-cell eQTL mapping. Notably, we demonstrate for the first time at the single-cell level the causal influence of mDCs markers on periodontitis. This research also enables the identification and validation of novel biomarkers and therapeutic targets, ultimately advancing the implementation of precision medicine in the management of periodontal disease.

## 2 Research methodology and data sources

### 2.1 Research design

**Figure 1:**
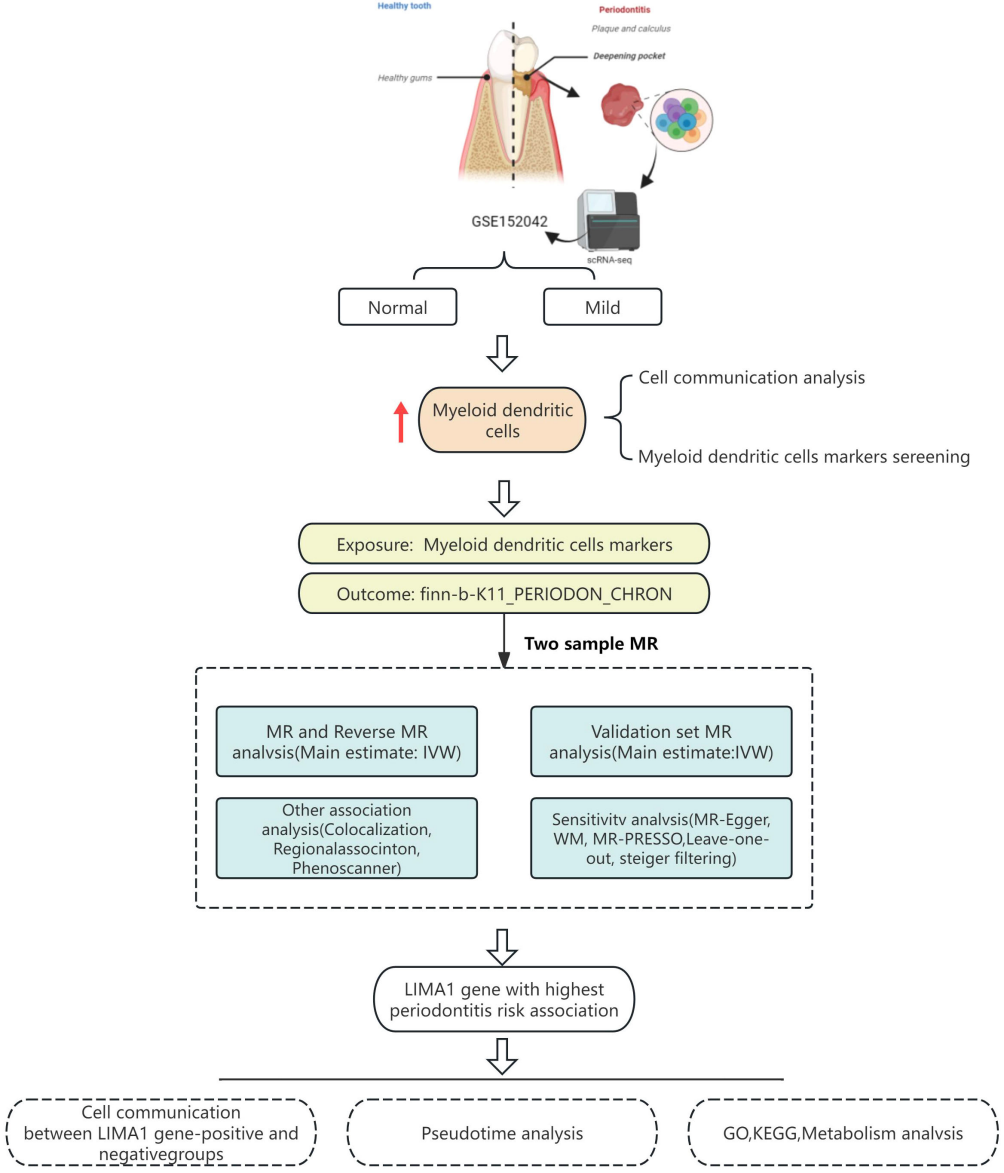
Schematic diagram of the research workflow.

This study obtained single-cell transcriptomic data from the Gene Expression Omnibus (GEO) database and analyzed it using R and the Seurat package. These tools facilitated data retrieval, quality control, dimensionality reduction, clustering, and annotation. Marker genes were employed to identify subpopulations within each cellular cluster. CellChat software was utilized to explore intercellular communication. MR analysis was performed to validate the eQTLs associated with the studied condition, utilizing single nucleotide polymorphisms (SNPs) to evaluate heterogeneity and pleiotropy for determining causal genes. A schematic diagram of the workflow is depicted in **Figure 1**.

### 2.2 Collection of sequencing data

The single-cell transcriptomic data utilized in this study was obtained from the GSE152042 dataset, which is accessible in the GEO database. This dataset comprises multiple samples, including samples from periodontitis patients and corresponding healthy controls. Data retrieval and analysis were conducted using R language and the Seurat package.

To ensure the integrity and accuracy of the data, a series of quality control standards were implemented. First, cells with fewer than 200 or more than 4,000 genes were excluded to eliminate damaged cells. Second, cells with mitochondrial gene expression accounting for more than 10% of total expression were removed to eliminate those in an abnormal state.

In the data preprocessing phase, the raw gene expression counts were first normalized to ensure that the total expression level for each cell equaled 10,000. Then, the NormalizeData function from Seurat was used to perform log transformation, stabilizing the variance between cells and adjusting for differences in sequencing depth. Next, the FindMarkers method was used to calculate the expression variance for each cluster, with a log2 mean difference in expression set at 0.5 and statistical significance at *p < 0.05*.

The data underwent scaling and centering, using the ScaleData function to ensure that the range of expression levels would not affect subsequent analyses. Principal component analysis (PCA) was then performed for dimensionality reduction, followed by uniform manifold approximation and projection (UMAP) for further dimensionality reduction. Finally, based on the PCA results, t-distributed stochastic neighbor embedding (t-SNE) was utilized for the visualization of cell clustering.

In studying the cell subpopulations in periodontal disease, the MonacoImmuneData was utilized, providing a comprehensive and validated set of immune cell phenotype markers that accurately identify and classify complex cell populations. The application of t-SNE and UMAP algorithms aids in preserving the overall structure of the data while highlighting clustering within the high-dimensional dataset, facilitating a more detailed interpretation of cellular functions and interactions.

This study primarily focuses on subpopulations such as monocytes and mDCs, that play crucial roles in immune responses and the inflammatory processes of periodontal tissue.

### 2.3 Single-cell RNA sequencing data analysis

The expression of marker genes facilitated the identification of significant subgroups within cellular populations related to periodontal disease. Initially, specific cells were isolated and analyzed through clustering. Following this, genes in each population underwent pseudotime and single-cell trajectory analysis. The communication network was inferred, analyzed, and visualized using the “CellChat” package in R. This tool utilizes gene expression data as input and groups cells by constructing a shared neighborhood graph based on distances in pseudotime trajectory space. CellChat then estimates the probabilities of intercellular communication by examining interactions between gene expression, signaling ligands, receptors, and other associated factors. Additionally, the “Viridis” package in R was employed to annotate the distribution of marker expression in the cell clusters. By analyzing the distinct features of various cell types, we can classify and metabolically evaluate cells, as well as identify alterations in Kyoto Encyclopedia of Genes and Genomes (KEGG) pathways related to the identified subgroups. We conducted Gene Ontology (GO) functional enrichment analysis and KEGG pathway enrichment analysis on the marker genes using the ClusterProfilerR software package.

### 2.4 Mendelian randomization validation of key gene eQTLs

To ensure the robustness and reliability of the findings, we followed the guidelines of the STROBE-MR checklist in conducting our MR analysis (13). Specifically, we identified 26 key markers for mDCs clusters by intersecting two different genomes and retrieved relevant eQTLs as exposure factors from the IEU Open GWAS project database (https://gwas.mrcieu.ac.uk/datasets/). Additionally, we accessed the periodontal disease cohort from this database, identified by the ID finn-b-K11_PERIODON_CHRON, which included 195,395 standard samples and 3,046 samples diagnosed with periodontal disease, providing whole-genome data that served as our outcome data. MR analysis was conducted on marker genes within each cluster and eQTLs associated with periodontal disease to identify causal genes relevant to the disease in each cluster.

Initially, we selected SNPs that were closely associated with gene expression, applying a significance threshold of *p < 5×10^−8^* when using marker genes as exposure factors. Subsequently, we computed the F-statistic to assess the strength of the association between instrumental variables and exposure factors. SNPs with an F-value less than 10 were excluded to mitigate potential weak instrument bias. The inverse variance-weighted fixed-effect (IVW-FE) model was employed as the primary MR analysis method. Cochran’s Q test was conducted to evaluate the heterogeneity among instrumental variables, with *p > 0.05* indicating a minimal likelihood of heterogeneity. MR Egger intercept tests were performed to assess horizontal pleiotropy, where a statistically significant intercept term would suggest the presence of significant horizontal pleiotropy.

In addition, we accessed the periodontitis cohort in the database through ID finn-b-K11_PERIODON_ACUTE to conduct MR analysis, further validating the causal relationship between the marker gene and periodontitis.

### 2.5 R-MR analyses

R-MR analyses were performed for all associations that survived multiple testing to investigate reverse causation (i.e., whether genetic predisposition to periodontitis influences the marker eQTL). To further validate this, the MR Steiger directionality test was employed to assess the directionality of the associations between marker eQTL and periodontitis.

## 3 Results

The workflow is visualized in **Figure 1**.

### 3.1 Data normalization and dimensionality reduction

**Figure 2:**
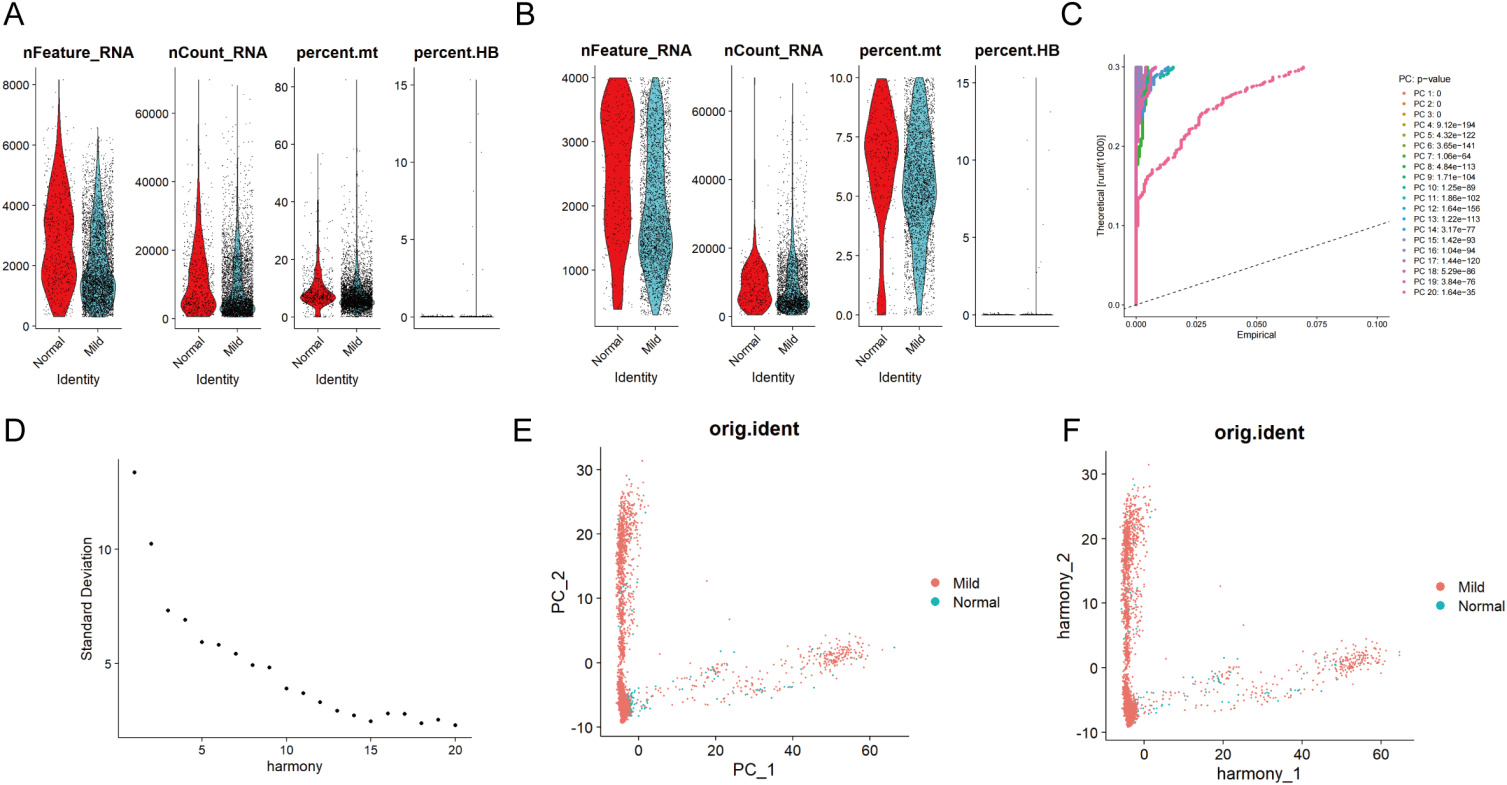
Single-cell quality control and standardization. Pre **(A)** and post-quality **(B)** control comparisons of nFeature_RNA, nCount_RNA, and percent mt in periodontal disease and control samples show improved data quality by removing low-quality cells and correcting sequencing depth discrepancies, enhancing analysis accuracy. **(C)** The principal component analysis method was used for dimensionality reduction on the integrated data, with the information (variance percentage) represented by each principal component ranked in percentage order, and a principal component line graph was generated to visualize the PCA downscaling. **(D)** In the fragmentation map for principal component screening, the x-axis represents the component number, and the y-axis shows the variance explained by each component. The first 15 PCs are selected based on the elbow point. **(E)** Distribution of cells in periodontal disease and control samples in principal components. **(F)** After eliminating batch effects, the correlation of gingival tissue cells between periodontal disease and normal control patients is shown in the PCA scatter plot.

The scRNA-seq and microarray datasets were obtained from the Gene Expression Omnibus (GEO) database. Additionally, the microarray datasets, including GSE152042 (platform: GPL16791), included data from gingival tissues of both periodontal patients and healthy individuals. The scRNA-seq analysis identifies all major cell types in gingival tissue, integrating results from periodontal disease-affected and healthy gingival tissues. After filtering out cells with fewer than 200 genes and genes detected in fewer than 3 cells, a total of 4,564 cells and 18,697 genes were retained (**Figure 2A**). Following a quality control step, the dataset was further refined to 3,544 cells while retaining the same number of genes (**Figure 2B**). Subsequently, the 15 principal components (PCs) contributing most significantly to the observed variation were identified (**Figures 2C, D**). Among these, the degree of clustering between samples in 2 PCs was relatively high (**Figures 2E, F**).

### 3.2 Single-cell transcriptomic analysis of periodontal disease

**Figure 3:**
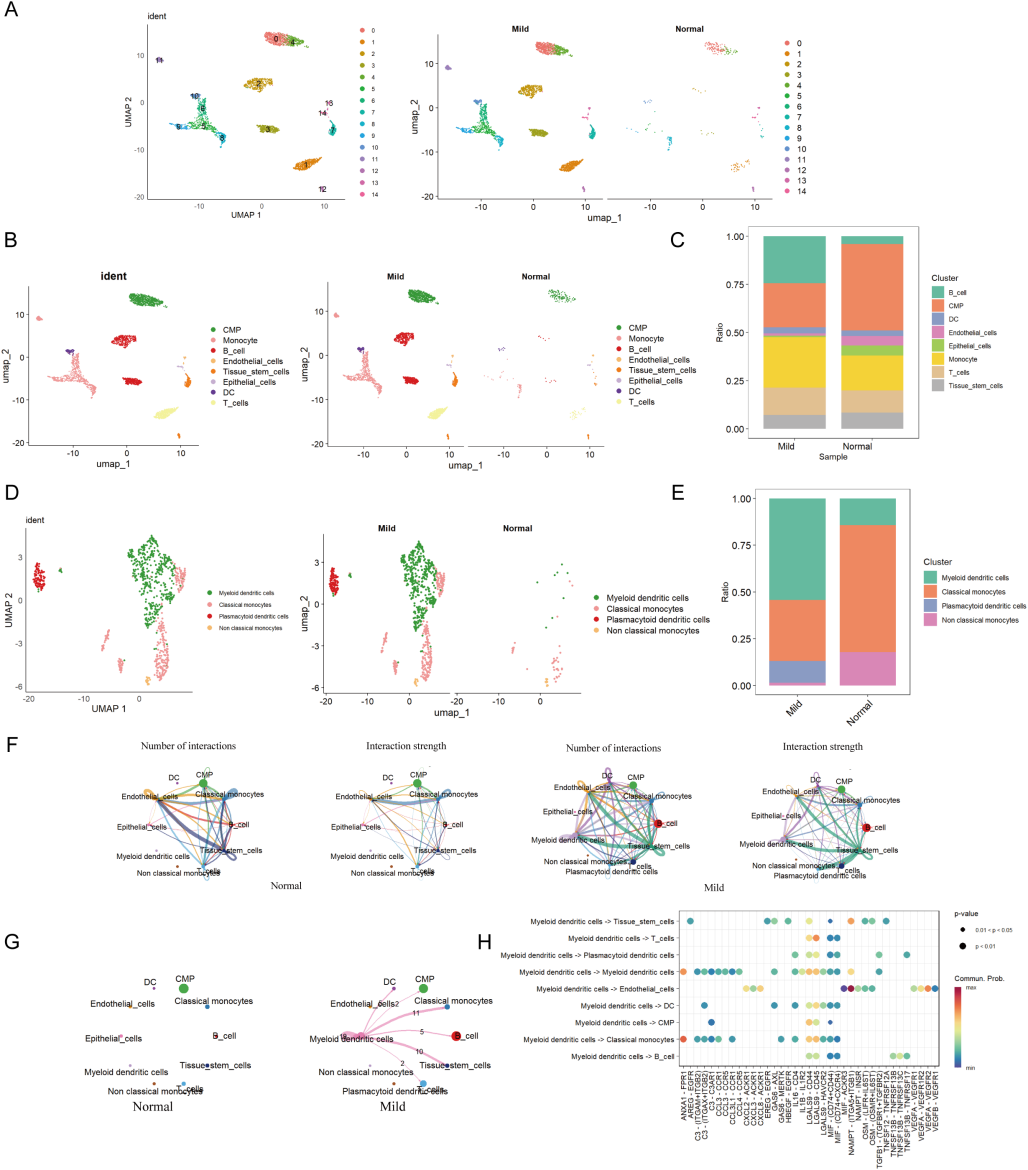
Cell clustering and communication analysis. **(A)** UMAP cell clustering of all samples in the single-cell dataset, with different colors representing distinct cell clusters. **(B)** Distribution of cell clusters in periodontal disease and control samples. **(C)** Percentage of different cell types in both periodontal disease and control groups. **(D)** Distribution of various cell types in periodontal disease and control groups. **(E)** Percentage of different cell types in both periodontal disease and control groups. **(F)** Number and strength of cell communication interactions in the periodontal disease group. Number and strength of cell communication interactions in the control group. **(G)** Number and strength of mDCs communication interactions in both the periodontal disease and normal control groups. **(H)** Proportion of communication interactions among mDCs.

In GSE152042, 14 distinct cell clusters were identified through UMAP cluster analysis (**Figure 3A**). Across all samples, 8 cell types were annotated via SingleR, namely CMP, Monocyte, B cell, Endothelial, Tissue stem cells, Epithelial cells, DC, and T cell (**Figures 3B, C**). By analyzing the expression patterns of immune cell marker genes, we identified four cell types: mDCs, Classical monocytes, Plasmacytoid dendritic cells, and non-classical monocytes. Dimensionality reduction plots and proportional representations were generated (**Figure 3D**). Notably, the levels of certain immune cells, particularly monocytes, were significantly elevated in patients with periodontal disease compared to healthy controls, indicating a strong correlation between monocyte levels and the severity of periodontal disease.

The most abundant population was mDCs, while the Monocyte population was predominantly comprised of Classical monocytes (**Figure 3E**). Moreover, there were no specific differences in cell populations between the control and periodontal groups. Among these, mDCs were most prevalent in the periodontal group, while these cells were less abundant in the control group (**Figure 3E**).

Furthermore, cell communication analysis among eight cell types in the two groups revealed that the periodontal disease group exhibited the most ligand-receptor relationships and the highest level of communication intensity (**Figure 3F**). The analysis indicated extensive interactions between mDCs and various other cell types, suggesting potential crosstalk between mDCs and a range of immune cells (**Figure 3G**). MDCs were found to communicate with T cells and classical monocytes via the LGALS9-CD45 pathway. Additionally, interactions between mDCs and classical monocytes were observed through pathways including ANXA1-FPR1, CCL3-CCR1, LGALS9-CD44, and LGALS9-CD45. They also interacted with B cells through MIF-(CD74+CD44) and MIF-(CD74+CXCR4) pathways (**Figure 3H**).

### 3.3 Utilization of MR to validate the causal relationship between key genes’ eQTL and periodontal

**Figure 4:**
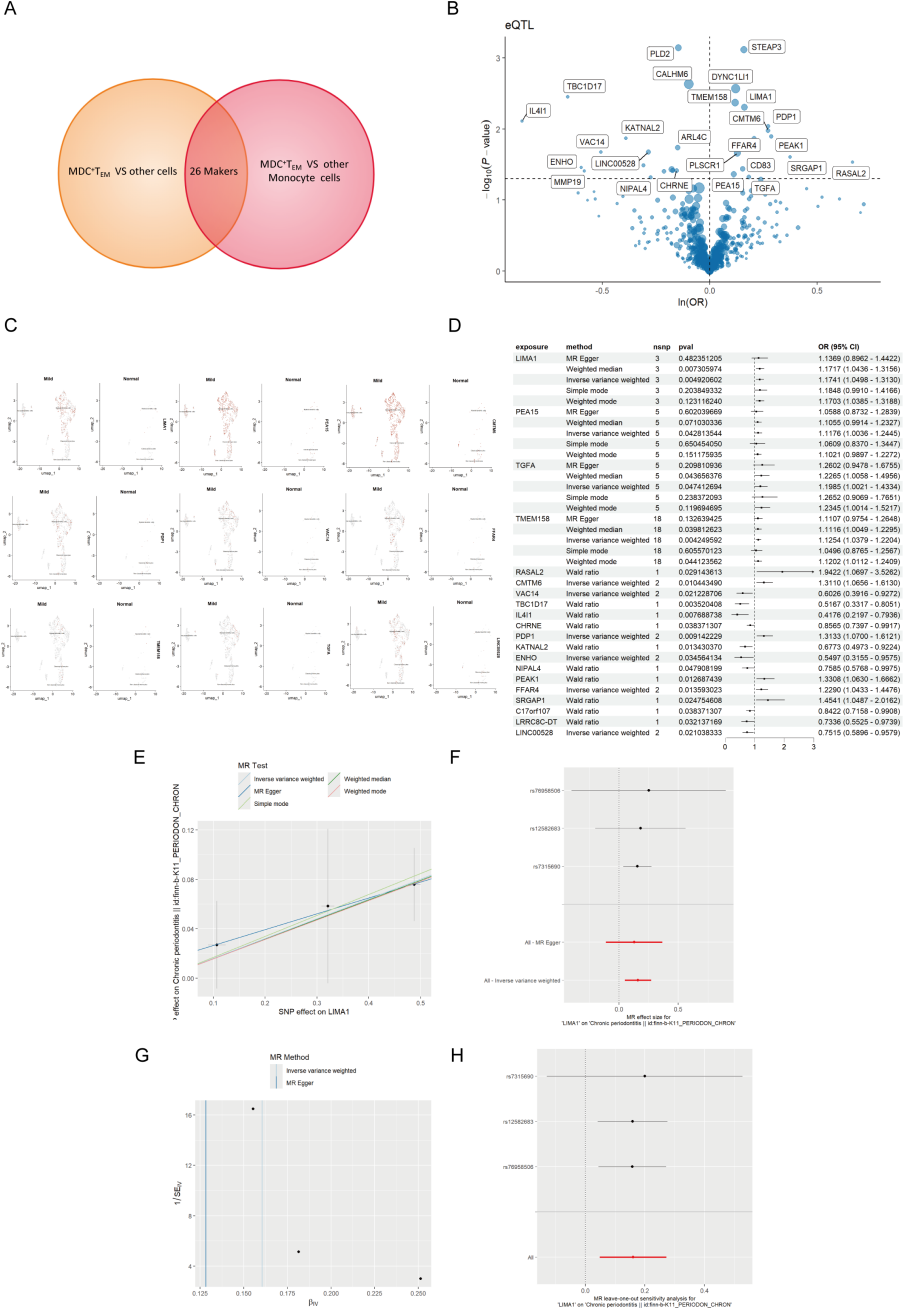
MR analysis of dendritic cells and periodontal disease. **(A)** The Venn diagram illustrates the dendritic cell cluster screening strategy, identifying 26 key markers in the mDCs cluster. **(B)** Volcano plot of MR results for multiple markers, showing the risk of periodontal disease determined using the IVW method. The odds ratios (ORs) for periodontal disease risk are represented by standard deviations of marker levels. **(C)** Expression levels of statistically significant marker (including LIMA1, PEA15, CMTM6, PDP1, VAC14, FFAR4, TMEM158, TGFA, and LINC00528) in single cells, as identified by the IVW method. **(D)** Forest plot of MR model results. **(E)** Scatter plot of the five MR models. Each point represents an IV, with the line on each point indicating the 95% CI. The y-axis shows the effect of SNPs on the outcome, and the x-axis shows the effect of SNPs on exposure. **(F)** Forest plot of MR analysis results estimated for the individual SNP LIMA1, with the red line representing the summary results of all SNPs, indicating that LIMA1 is a risk factor. **(G)** Funnel plot of three SNPs on MR analysis. **(H)** MR sensitivity analysis for LIMA1 after removing SNPs using the leave-one-out method. The red line represents the pooled results for all SNPs.

eQTL data were sourced from the IEU OpenGWAS project (https://gwas.mrcieu.ac.uk/). The periodontal GWAS data from FinnGen R11 (https://www.finngen.fi/fi) encompassed data from 13,261 periodontal patients and 195,395 healthy individuals, all derived from individuals of European ancestry. To investigate the genetic relationship between mDCs and periodontal disease, we selected markers of mDCs and analyzed them using MR through a genome-wide association study (GWAS). Initially, cross-analysis of data from two distinct genomes identified 26 key markers from mDCs clusters (**Figure 4A**), along with volcano maps of marker genes (**Figure 4B**) and their expression at the single-cell level (**Figure 4C**). The study found that LIMA1 demonstrated a strong correlation with the risk of periodontitis at the single-cell level, with expression levels significantly higher in patients with periodontitis compared to healthy individuals.

Mapping eQTL is an effective approach for examining how common genetic variations influence gene expression among individuals (14). To explore the role of these markers in periodontitis, a total of 96 eQTLs linked to the expression of 26 markers were identified following the clustering of SNPs in linkage disequilibrium (*r² < 0.001*). The average F statistic for the SNPs employed as instruments ranged from 30.17 to 3,141.78, indicating strong instrumental variables. Public genome-wide association study data were utilized in a two-sample MR analysis, wherein eQTL SNPs served as instrumental variables, the markers functioned as exposure variables, and periodontitis represented the outcome variable. The MR analysis revealed that ten markers related to mDCs possessed a direct causal link to the onset of periodontitis. Specifically, *LIMA1* (IVW, 3 SNPs, *p = 0.004*), *PEA15* (IVW, 5 SNPs, *p = 0.04*), *TGFA* (IVW, 5 SNPs, *p = 0.04*), *TMEM158* (IVW, 18 SNPs, *p = 0.004*), *CMTM6* (IVW, 2 SNPs, *p = 0.01*), *PDP1* (IVW, 2 SNPs, *p = 0.009*), and *FFAR4* (IVW, 2 SNPs, *p = 0.01*) were identified as risk factors for periodontitis, while *VAC14* (IVW, 2 SNPs, *p = 0.02*) and *ENHO* (IVW, 2 SNPs, *p = 0.03*) were recognized as protective factors (**Figure 4D**). Additionally, we generated scatter, forest, funnel, and leave-one-out plots (**Figures 4E–H**) for further analysis of LIMA1.

**Figure 5:**
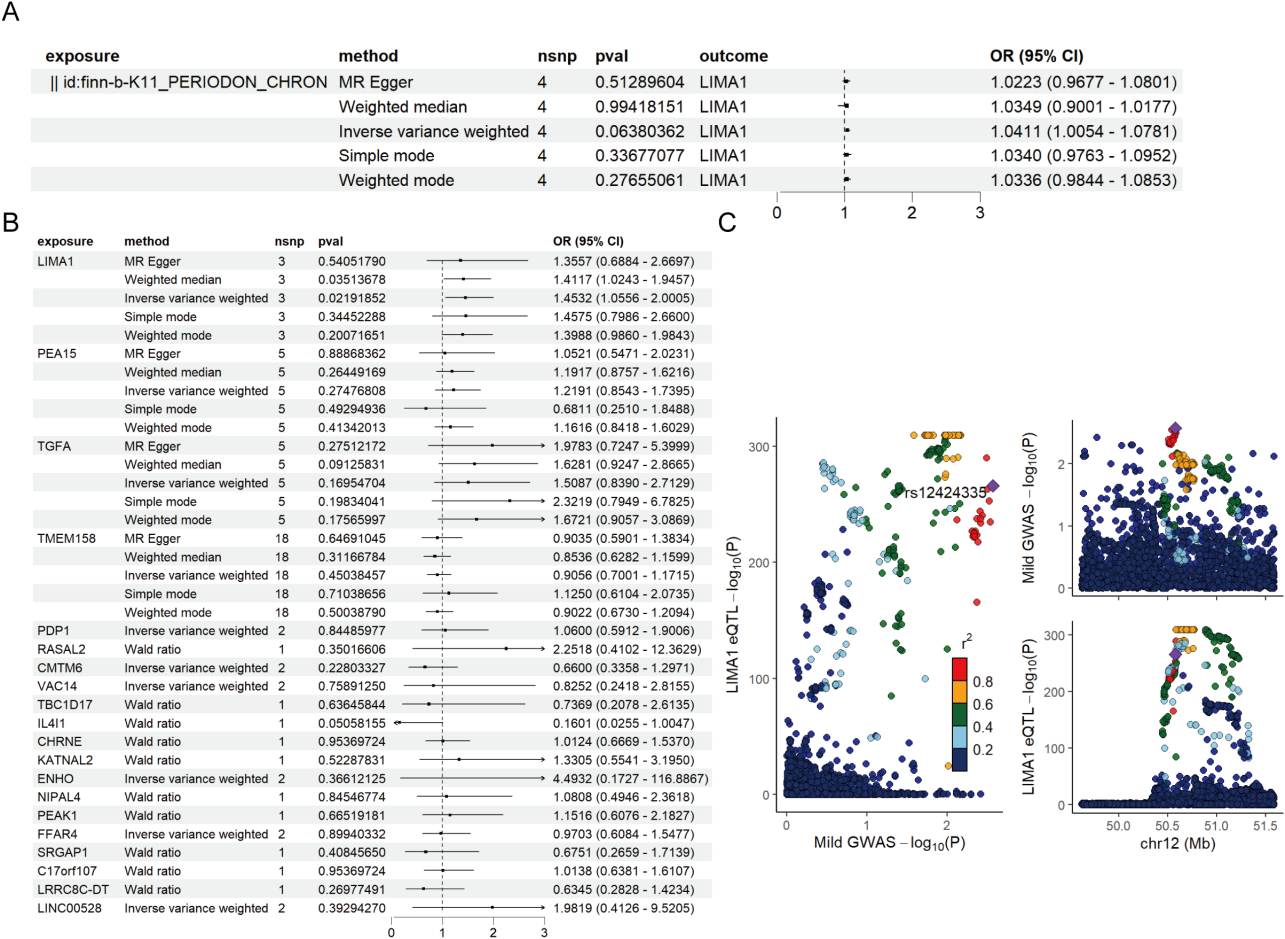
Mendelian randomization validation analysis and reverse Mendelian randomization between marker genes and periodontal disease. **(A)** Validation of the reverse Mendelian randomization analysis between periodontal disease and LIMA1 indicates no reverse causal relationship. **(B)** Forest plot of MR validation through finn-b-K11_PERIODON_ACUTE. **(C)** Regional association plot of GWAS results and marker-eQTLs at the marker, PTB, and PTB locus. SNPs are colored based on LD (r2) with the lead marker-eQTL (rs12424335). Purple diamonds represent the lowest p-value for each locus.

For MR validation, the periodontal validation set ID “finn-b-K11_PERIODON_ACUTE” confirmed the reliability of LIMA1, showing no evidence of heterogeneity (all *p > 0.1*), pleiotropy (all *p < 0.05*), or reverse causality (all *p > 0.05*) (**Figure 5B**). Among all SNPs for LIMA1, the most significant risk factor for periodontal disease was rs7315690, which exhibited the highest F statistic and the lowest p-value for correlation with *LIMA1* (*F = 1576.22, p = 4.92 × 10^-3^*). Additionally, we conducted reverse MR analysis, and the results indicated no evidence of reverse causality, suggesting that there is no inverse relationship with LIMA1 (**Figure 5A**).

Finally, we performed a co-localization analysis of these genes at the eQTL-GWAS level. Our results indicated that LIMA1 is associated with periodontal disease and shares genetic loci with specific mutations (**Figure 5C**).

### 3.4 Analysis of single-cell RNA sequencing data of marker gene

**Figure 6:**
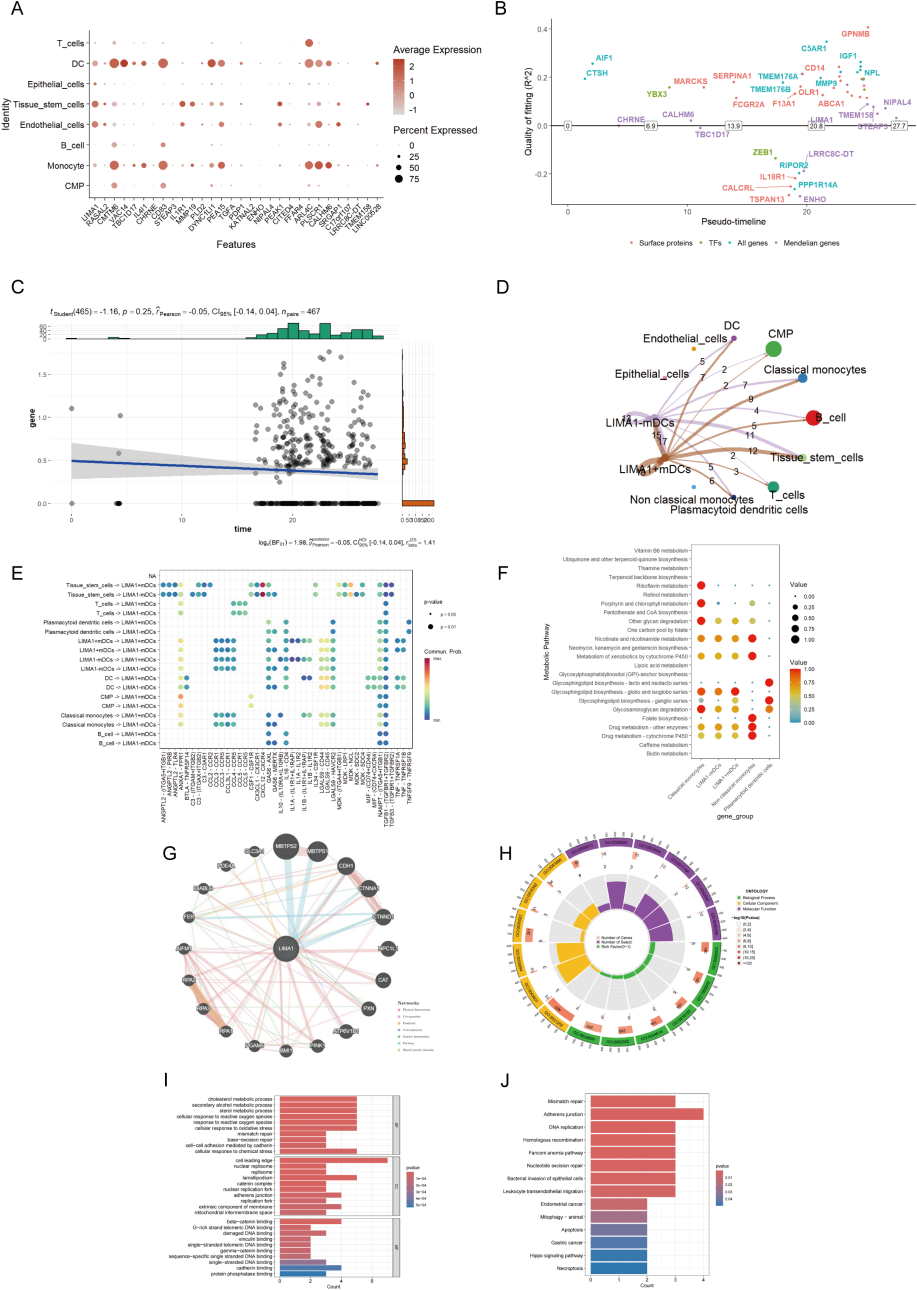
Core downstream functional analysis of dendritic cell clusters marked in periodontitis. **(A)** Proportions of marked gene expression across different cell types. **(B)** Pseudotime analysis of marked genes. **(C)** Correlation analysis between marked genes and the progression of periodontitis. **(D)** The number of receptor-ligand pairs shown by the intercellular communication network between LIMA1-positive and -negative dendritic cells and other cell subpopulations. The thickness of each line represents the strength of paired interactions. **(E)** Bubble plot of possible interaction pathways between LIMA1+ mDCs, LIMA1- mDCs, and other cells in periodontitis patients. **(F)** Bubble chart of enriched metabolic pathways, highlighting metabolic differences between LIMA1-positive and -negative dendritic cells. Each bubble represents a metabolic pathway, with p-values and the total number of metabolites involved listed on the right. **(G)** Analysis of gene-gene interaction networks for biomarkers using the GeneMANIA database. **(H, I)** Description of marked gene GO analysis using fanyiwei. **(J)** Bar chart showing KEGG analysis of marked genes.

Our study elucidated the causal relationship between 26 identified marker genes and periodontal disease. We visualized the expression levels of these genes at the cellular level, including B cells, T cells, monocytes, and endothelial cells (**Figure 6A**).

Subsequently, we conducted a developmental trajectory visualization analysis of these marker genes at the monocyte level. The results demonstrated that LIMA1 functions as a downregulation switch gene in periodontal disease, with the expression profiles of their surface proteins and associated transcription factors undergoing significant changes over time, indicating their crucial role in monocyte development (**Figure 6B**). Furthermore, correlation analysis between the expression of the marker genes and periodontal progression suggested a negative correlation with expression levels over time (**Figure 6C**).

Next, we investigated cell communication and cellular metabolic pathways between mDCs and LIMA1-positive and negative groups. The results indicated that communication intensity levels in LIMA1-positive bone marrow mDCs were higher compared to the negative group, reflecting extensive interactions with various other cell types (**Figure 6D**). This LIMA1-positive group also exhibited enhanced ANXA1-FPR1 and CXCL12-CXCR4 pathways in terms of cell communication compared to the LIMA1-negative mDCs group (**Figures 6E**). Differential expressions were observed at the cellular metabolism level in pathways including other glycan degradation, Glycosphingolipid biosynthesis-ganglio, and Glycosphingolipid biosynthesis-globo and isoglobo series (**Figure 6F**). Additionally, the LIMA1-positive group displayed an increase in the expression of Glycosphingolipid biosynthesis-ganglio, suggesting a stronger correlation.

LIMA1 is present in various network types within the protein-protein interaction (PPI) network, including co-expression, co-localization, and genetic interactions (**Figure 6G**). Utilizing the ClusterProfilerR package, we conducted GO and KEGG enrichment analyses on the marker genes. The GO analysis encompassed biological processes (BP), cellular components (CC), and molecular functions (MF). The enriched BP terms included cellular responses to reactive oxygen species. The enriched CC terms, such as the leading edge of cells and lamellipodia, indicate that LIMA1 may uphold the stability and functionality of lamellipodia by regulating actin polymerization and depolymerization, thereby facilitating cellular motility. Furthermore, the enriched MF terms involved beta-catenin binding, single-stranded DNA binding, and binding to damaged DNA (**Figures 6H, I**).

The KEGG pathway enrichment analysis identified significant pathways, including bacterial invasion of epithelial cells, leukocyte transendothelial migration, and apoptosis in the context of periodontitis. This implies that these biological processes may play crucial roles in the onset and progression of periodontitis (**Figure 6J**).

## 4 Discussion

This study seeks to elucidate the molecular mechanisms underlying periodontal disease through scRNA-seq and eQTL analysis, thereby providing novel insights into its etiology. We identified a significant subset of cells—mDCs—within the gingival tissue of patients with periodontal disease, which exhibited specific functional characteristics. By analyzing existing genome-wide association study (GWAS) data, we assessed the potential causal impact of mDCs markers on the risk of periodontal disease. Our findings demonstrate that mDCs markers play a pivotal role in the pathogenesis of periodontal disease, identifying several key markers, such as LIMA1, as potential targets for future preventive and therapeutic strategies. This research contributes essential evidence for a deeper understanding of the immune mechanisms involved in periodontal disease and opens new avenues for related intervention strategies.

By utilizing scRNA-seq data and MR techniques, our study analyzed the cellular and molecular characteristics of periodontitis. Through dimensionality reduction and clustering, we identified four distinct cell phenotypes. Compared to the control group, the monocyte phenotype was significantly elevated in patients with periodontitis, enhancing our understanding of the role of monocytes in the pathophysiology of the disease. Further investigation into the monocyte subpopulations revealed significant disparities in mDCs expression levels between patients with periodontitis and healthy individuals, suggesting their potential as biomarkers or therapeutic targets. Additionally, our analysis of cell communication indicated that the ligand-receptor interactions among patients with periodontitis were notably complex, with the highest communication intensity observed. This complexity underscores the critical role of mDCs in modulating the immune response to periodontitis. The relationship between mDCs and periodontitis is mediated through various mechanisms, including inflammatory responses, immune modulation, and intercellular signaling (15–17). As a crucial component of the immune system, mDCs are responsible for presenting antigens from external pathogens to naïve T cells, thereby initiating and amplifying the immune response (18–20). Specifically, mDCs capture, process, and present antigens, activating and promoting T cell proliferation—a process essential for generating effective immune responses against periodontal pathogens (21, 22). However, dysfunction in the maturation and regulatory capacities of mDCs may lead to inappropriate immune responses. Such dysregulation can trigger excessive inflammatory reactions, resulting in tissue damage and exacerbating the progression of periodontitis (23, 24). For instance, when mDCs fail to adequately regulate immune responses, they may attack host tissues, leading to pathological consequences (25, 26). Moreover, mDCs are particularly susceptible to periodontal pathogens, such as Porphyromonas gingivalis; their invasion can impair mDCs function, diminishing their efficacy in responding to immune challenges, thereby accelerating the deterioration of periodontitis (27–29). The distribution and function of mDCs in the oral mucosa are also noteworthy. They are not only present in the gingival epithelium and connective tissue but also play a role in monitoring and responding to periodontal pathogens. MDCs are indispensable for maintaining periodontal health, regulating immune responses, and defending against pathogen invasion, thereby ensuring the stability and health of the oral environment. Therefore, an in-depth investigation of mDCs functions is crucial for understanding the pathological mechanisms of periodontitis.

MR analysis corroborated the inferences drawn from scRNA-seq, identifying ten causal gene markers associated with periodontitis, including LIMA1, PEA15, TMEM158, CMTM6, PDP1, FFAR4, VAC14, and ENHO. LIMA1 exhibited the strongest association with periodontal disease risk, underscoring its significance in the disease’s pathogenesis. In periodontitis patients, the odds ratio (OR) for LIMA1 was 1.17 (95% confidence interval (CI): 1.04–1.31), suggesting that LIMA1+ mDCs patients are 1.17 times more likely to develop periodontitis compared to LIMA1− mDCs patients. Our findings were further validated using independent cohorts, ensuring the reliability of the results across different populations. Functional enrichment analysis revealed that these gene markers are involved in critical biological processes, including responses to reactive oxygen species. LIMA1, also known as the epithelial protein, lost in tumors (EPLIN) and sterol regulatory element-binding protein 3 (SREBP3), was initially identified as a differentially expressed gene in oral epithelial carcinogenesis through cDNA differential analysis. Subsequently, maul et al. first described and confirmed the existence of LIMA1 as a novel cytoskeletal protein. LIMA1 has two distinct isoforms: LIMA1-a, which consists of 600 amino acids, and LIMA1-b, which comprises 759 amino acids. As a LIM domain protein, LIMA1 provides binding sites for specific signaling proteins and facilitates its own dimerization or interaction with other proteins. Due to its importance in regulating actin cytoskeletal dynamics and its potential involvement in cadherin-mediated cell adhesion, the loss of LIMA1 in cancer cells may affect cellular behavior, enhancing their invasive characteristics (30). LIMA1 deficiency could lead to dysregulation of cytoskeletal dynamics, altered motility, and impaired intercellular adhesion, thereby promoting tumor proliferation, invasion, and migration (31, 32).

In summary, the elevated expression of LIMA1 in patients with periodontitis suggests its potential as a diagnostic biomarker, which could aid in the early detection of the disease. Additionally, targeting the LIMA1 pathway may offer a promising therapeutic approach to modulate immune responses associated with periodontitis. LIMA1 may also have utility in risk stratification, helping to guide personalized treatment strategies. Further studies are needed to assess the clinical applicability of LIMA1, including its role in disease progression monitoring and treatment response.

## 5 Conclusion

This study highlights the role of mDCs and their markers, particularly LIMA1, in the pathogenesis of periodontal disease. Through the integration of single-cell RNA sequencing and Mendelian randomization, we identified several key genetic markers that contribute to disease progression, with LIMA1 showing a strong association with periodontitis risk. Our findings suggest that LIMA1 could be a potential diagnostic biomarker and a therapeutic target for personalized treatment strategies. While these results provide new insights into the immune mechanisms underlying periodontal disease, further studies with larger sample sizes and experimental validation are needed to confirm these findings and fully explore the clinical potential of mDCs and their markers.

## Data Availability

The GWAS data used in this study are publicly available, and their origins are described appropriately in the manuscript (https://gwas.mrcieu.ac.uk). The FinnGen data can be accessed via this website (https://www.finngen.fi/en/access_results). The datasets generated and analyzed in this study are available in the GEO data repository (https://www.ncbi.nlm.nih.gov/geo): GSE152042.
